# Evaluating multi-task network architectures for simultaneous breast lesion segmentation and classification in ultrasound images

**DOI:** 10.1007/s11517-026-03592-2

**Published:** 2026-05-15

**Authors:** Margarida R. Ferreira, Helena R. Torres, Bruno Oliveira, Augusto R. V. F. de Araújo, Pedro Morais, Paulo Novais, João L. Vilaça

**Affiliations:** 1 2Ai – School of Technology, IPCA, R. de São Martinho, 4750-810 Vila Frescainha (São Martinho), Barcelos, Portugal; 2 Algoritmi Center, School of Engineering, University of Minho, Guimarães, Portugal; 3 LASI - Intelligent Systems Associate Laboratory, Guimarães, Portugal; 4 DU Robotics, Maersk McKinney Moller Institute, University of Southern Denmark, Odense, Denmark

**Keywords:** Breast ultrasound, Classification, Deep learning, Multi-task learning, Segmentation

## Abstract

Breast lesion segmentation and classification in ultrasound (US) images are two essential tasks for computer-aided diagnosis of breast cancer. However, these tasks are still challenging, mainly due to the high variability of lesions and the poor image quality of US. Numerous deep learning methods have been proposed to assist physicians in performing breast lesion segmentation and classification. Considering that these tasks are related to common features, learning them jointly through multi-task learning (MTL) represents a viable approach to improve the performance of each individual task. In this paper, we present and compare multiple MTL network configurations for the simultaneous segmentation and classification of breast lesions in ultrasound images. Building on two state-of-the-art architectures, namely SegResNet for segmentation and EfficientNet for classification, we designed and evaluated several combined configurations of these models arranged in different MTL schemes. These configurations explore various levels of feature sharing and integration between the tasks, aiming to identify the most effective architectural arrangement for joint lesion segmentation and malignancy classification in breast ultrasound. Moreover, these configurations are also compared with state-of-the-art MTL configurations. Experimental results based on a dataset compiled from two different centers, comprising 810 2D breast US images, indicate that combining SegResNet and EfficientNet in a MTL configuration achieved accurate performance in both segmentation and classification. Among the tested configurations, the best-performing model combined a segmentation network with an EfficientNet classification network that utilized shared encoded features from the SegResNet, achieving a Dice coefficient of 81.19% for lesion segmentation and an area under the ROC curve (AUC) of 97.27% for lesion classification. Overall, the proposed networks demonstrated their potential in enhancing computer-aided diagnosis of breast cancer.

**Graphical abstract:**

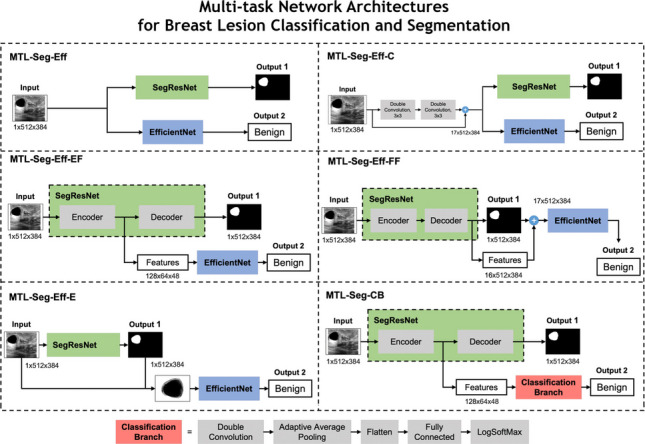

## Introduction

Breast cancer is the most common cancer in the world, affecting mainly women and having the highest cancer mortality among them [1]. According to the Global Cancer Observatory statistics, in 2020, there were nearly 2.3 million newly diagnosed cases and 685,000 deaths from breast cancer [1]. To counter this, diagnosis at an early stage is key for reducing the mortality rate as more efficient treatments can be provided [2]. Here, Ultrasound (US) is a widely used imaging modality for breast cancer screening as it is portable, cost-efficient, radiation-free, and real-time [3]. However, the interpretation and acquisition of US images is highly observer-dependent and requires experienced and well-trained physicians due to the presence of speckle, low signal-to-noise ratio, and shadows in US images, as well as the significant breast tumor variability [4]. This has motivated the development of computer-aided diagnosis (CAD) systems to assist radiologists in interpreting the US images [5].

Tumor identification/segmentation and classification are two basic tasks in breast cancer CAD systems [2]. Various methods have been explored for these tasks, notably convolutional neural networks (CNNs), have been explored to aid clinical practice [6]. On one hand, in our previous work [7], the performance of several state-of-the-art CNNs was compared for lesion segmentation task in breast US images, where a SegResNet-based architecture [8] outperformed other models while requiring fewer computational resources. On the other hand, many classification networks have been proposed and compared, showing remarkable results in medical image classification [9]. Particularly, EfficientNet, proposed by Tan and Le, achieved better accuracy and efficiency than previous CNNs [10]. In fact, in our previous comparative study [11], EfficientNet showed improved performance for breast lesion classification when compared with other classification networks.

The studies performed in [7] and [11] identified the most effective CNN models for segmentation and classification of breast US images, corroborating that CNNs are well-suited for these tasks. However, challenges persist, especially when dealing with low-contrast images, noise, or lesion variability. Multi-task learning (MTL), which trains a single model to perform related tasks simultaneously, offers a promising approach by promoting shared feature representations and improving generalization [12]. In fact, segmentation and classification are closely related tasks, and in clinical diagnosis, lesion appearance, boundary definition, and surrounding tissue characteristics are valuable for both and classification [13]. Thus, jointly training segmentation and classification within an MTL framework enables effective feature sharing between tasks, often leading to improved performance.

MTL has been applied in numerous medical image analysis tasks [13–16]. In 2019, Le et al. proposed an MTL architecture, which combines pixel-level segmentation and global image-level classification annotations for cancer diagnosis using mammograms [14]. Using a ResNet backbone, this network is based on a fully convolutional network (FCN), which allows efficient feature sharing between image regions and fast prediction. The classification branch takes the shared representation as input and outputs the predicted class (benign or malignant). In 2021, Zhou et al. proposed an MTL method for tumor segmentation and classification in 3D automated breast US [13]. The method employs a V-Net [17] backbone and consists of two sub-networks: an encoder-decoder network for segmentation and a lightweight multi-scale network for classification. A classification branch was added to the bottom of the V-Net, which receives fused feature maps from different stages. It contains two fully connected (FC) layers and one softmax layer to predict the input volume as benign or malignant. In 2022, Kang et al. proposed a multi-stage MTL network for thyroid nodule diagnosis, which performs three tasks in two stages: binary segmentation (background or nodule) and classification (benign or malignant) in the first stage, and multi-class segmentation in the second stage [15]. Intra and inter-task consistent learning was employed to enforce the network learn consistent predictions for all the tasks. The first stage uses a MTL U-Net with a classification branch after the encoder. The classification branch consists of one adaptive average pooling layer, one flatten layer, one FC layer, and one softmax layer. In the second stage, another U-Net is used to perform 3-class segmentation (background, benign, or malignant nodule). Furthermore, the same year, Chowdary et al. introduced a MTL method for breast lesion segmentation and classification in US images, that consists in a Residual U-Net with a classification branch [16]. This classification branch concatenates the extracted features from the last block of encoder, bridge and the first block of the decoder using a global average pooling layer and contains two FC layers with a dropout layer in between, and one softmax layer. This model is evaluated on the BUSI dataset. The authors of these four studies verified that MTL boosts the performance of both tasks. Although many MTL networks have been investigated, this paradigm has not yet been applied to a large multi-center dataset of breast US images.

In this paper, we present a comprehensive evaluation of MTL configurations for simultaneous breast lesion segmentation and classification in US images. Building on previous findings [7, 11], SegResNet and EfficientNet were selected as backbone architectures for segmentation and classification, respectively. Several MTL architectures were designed by combining these models in different configurations, varying levels of feature sharing and task integration, to identify the most effective arrangement for improving both tasks. The goal is to investigate how different architectural designs combining high-performing task-specific networks influence task synergy within an MTL framework, and whether these custom configurations combining task-effective segmentation and classification networks outperform existing state-of-the-art MTL architectures. The source code for the different MTL architectures is available at: https://github.com/2Ai-Laboratory/Breast_MTL.

The major contributions of the paper are listed as follows:

1. We explore the MTL paradigm for breast tumor diagnosis by designing MTL networks that jointly perform segmentation and classification of breast lesions in US images. The proposed networks incorporate two state-of-the-art networks, namely SegResNet and EfficientNet, tailored to each individual task;
2. We evaluate and compare multiple MTL network configurations based on different levels of feature sharing and architectural integration, identifying the most effective design for enhancing both tasks;
3. We benchmark the proposed configurations against state-of-the-art MTL models to assess their relative performance in terms of accuracy;
4. We validate all models on a dataset compiled from two independent breast US datasets acquired from different clinical centers, ensuring robustness and generalizability of the results.

The paper is organized as follows. In Section 2, a detailed description of the proposed network configurations is provided. Section 3 describes the breast US dataset, the evaluation metrics, and the implementation details. Section 4 presents the results from the evaluation of different MTL configurations, along with a comparison against state-of-the-art MTL methods. In Section 5, the results are discussed. Finally, the main conclusions are given in Section 6.

## Methods

In this section, the individual networks used for segmentation and classification are first described (Section 2.1), providing the foundation for the subsequent MTL architectures. Next, we introduce the proposed MTL models (Section 2.2), which combine and integrate the individual segmentation and classification networks for processing 2D breast US images. Finally, Section 2.3 details the loss functions employed across all MTL configurations. These MTL architectures combine both tasks into a single end-to-end CNN that takes a breast US image as input and simultaneously outputs a predicted segmentation mask and classification label (benign or malignant).

### Single-task networks for segmentation and classification

**Fig. 1:**
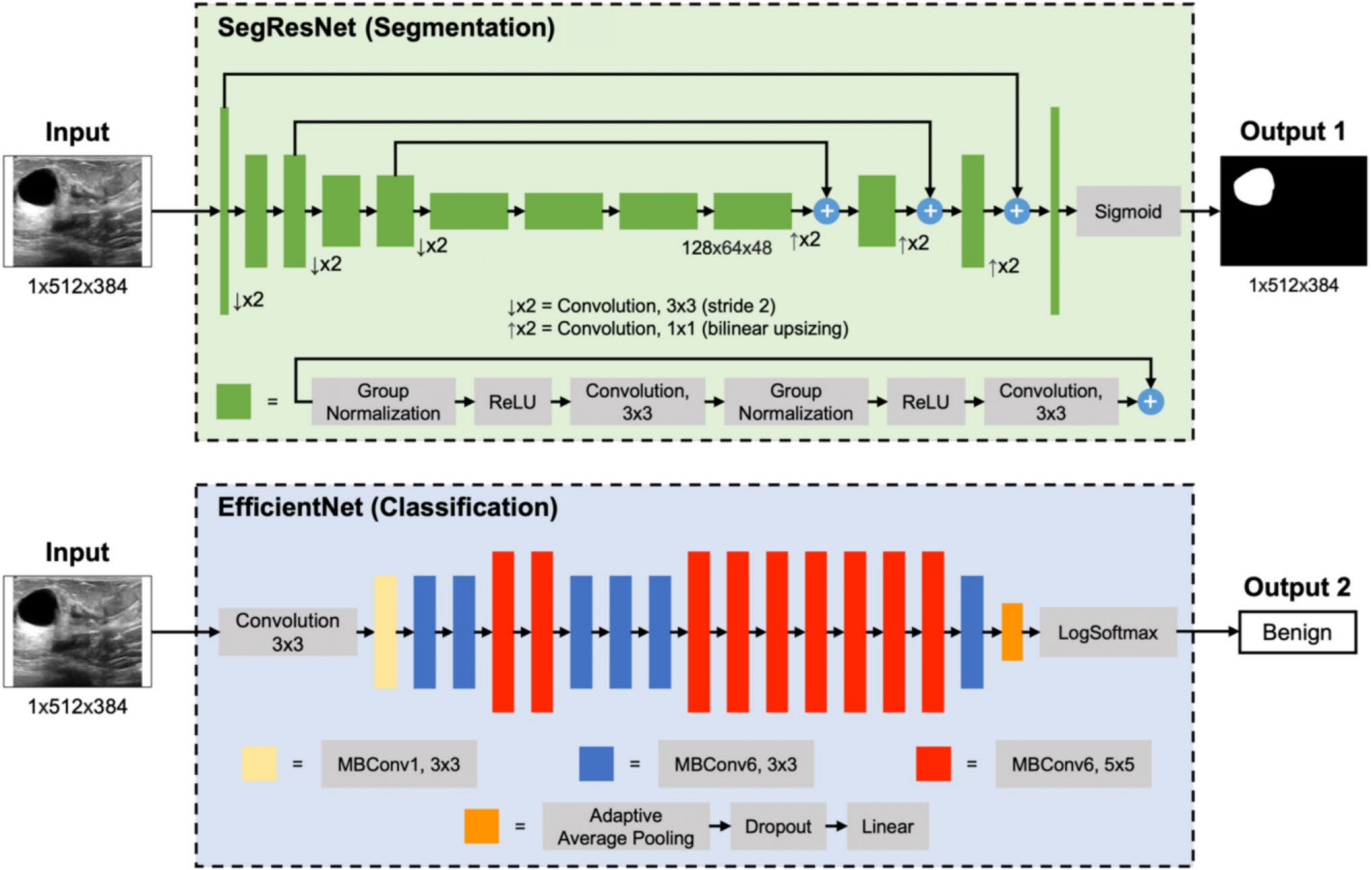
SegResNet and EfficientNet architectures for breast lesion segmentation and classification, respectively

To identify the optimal architectures for both segmentation and classification task, the literature was carefully evaluated, and comparative studies were performed regarding segmentation and classification networks for breast lesion analysis in US [7, 11]. In our previous comparative study [7], we concluded that GG-Net and SegResNet-based architectures were the best options for breast lesion segmentation in US images. In particular, SegResNet obtained the highest accuracy, while requiring low computational resources. Therefore, the SegResNet architecture is used for the segmentation task in the evaluation performed in this work. One comparative study was also performed by the authors regarding classification networks from breast lesion classification [11]. In this work, it was observed the high accuracy of the state-of-the-art EfficientNet for classification tasks, corroborating the results published in other studies [9, 10]. Thus, following our previous studies, we employed the state-of-the-art SegResNet for segmentation and EfficientNet for classification, integrating them into a multitask framework. The architectures for SegResNet and EfficientNet are illustrated in Fig. 1, while further details being given in the next sebsections.

#### Segmentation

SegResNet combines elements of a ResNet [18] with a U-Net [19] architecture [8]. It follows an asymmetrical encoder-decoder-based architecture, where the encoder is larger than the decoder. The encoder extracts image features, and the decoder reconstructs the segmentation mask. The encoder part employs ResNet blocks that consist of two convolutions with Group Normalization and ReLU, followed by an additive identity skip connection. The image dimensions are progressively reduced by 2 using strided convolutions, and simultaneously the feature size is increased by 2. All convolutions are 3 × 3 filters, with 16 channels in the first layer, selected for optimal performance compared to 8 or 24 initial filters. The decoder has a single ResNet block per each spatial level. In each level, the image dimensions are up-sized. Here, the number of features is reduced by a factor of 2 through 1 × 1 convolutions, and the spatial dimension is doubled using bilinear upsampling, followed by the addition of the encoder output of the equivalent spatial level. The decoder is followed by a 1 × 1 convolution, which generates a 1-channel image and a Sigmoid as the activation function. A variational autoencoder branch is also proposed in [8], but was not optimized in this work to prioritize segmentation and classification.

#### Classification

**Fig. 2:**
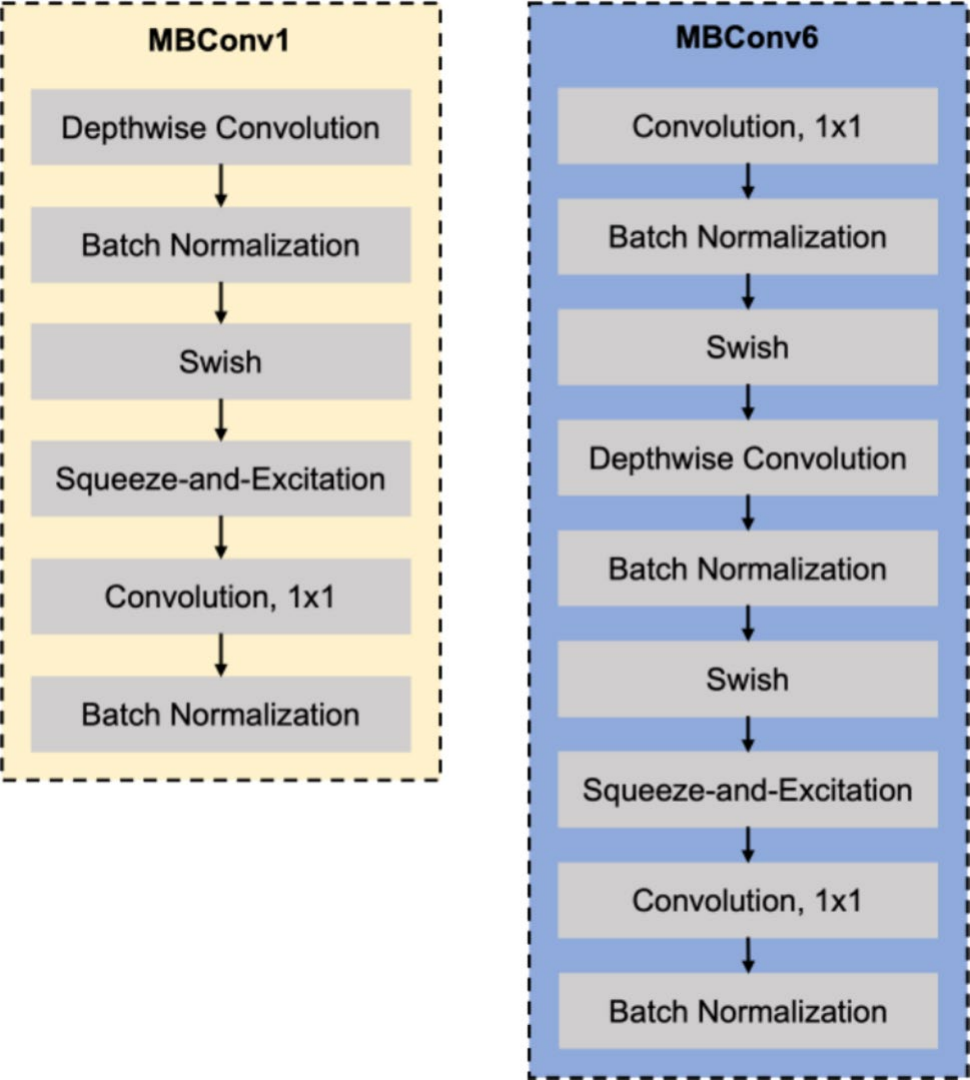
Structure of MBConv1 and MBConv6 that constitute EfficientNet

EfficientNet-B0, used for the classification task, is the baseline network of the EfficientNet group of CNN models. EfficientNet has been shown to achieve state-of-the-art accuracy on a variety of computer vision tasks while using significantly fewer parameters and less computation than previous models [10]. It is inspired by MobileNetV2, using mobile inverted bottleneck MBConv as the main building block. MBConv building blocks with 3 × 3 and 5 × 5 depthwise convolutions, squeeze-and-excitation, and swish activation. Figure 2 illustrates the structure of MBConv1 and MBConv6 layers. The EfficientNet architecture consists in a 3 × 3 MBConv1, two 3 × 3 MBConv6, two 5 × 5 MBConv6, three 3 × 3 MBConv1, seven 5 × 5 MBConv6, followed by a 3 × 3 MBConv6. After, one adaptive average pooling layer, one dropout layer, and a linear layer are added. LogSoftmax activation function is used to obtain the probability of membership for each class.

### Multi-task learning architectures

**Fig. 3:**
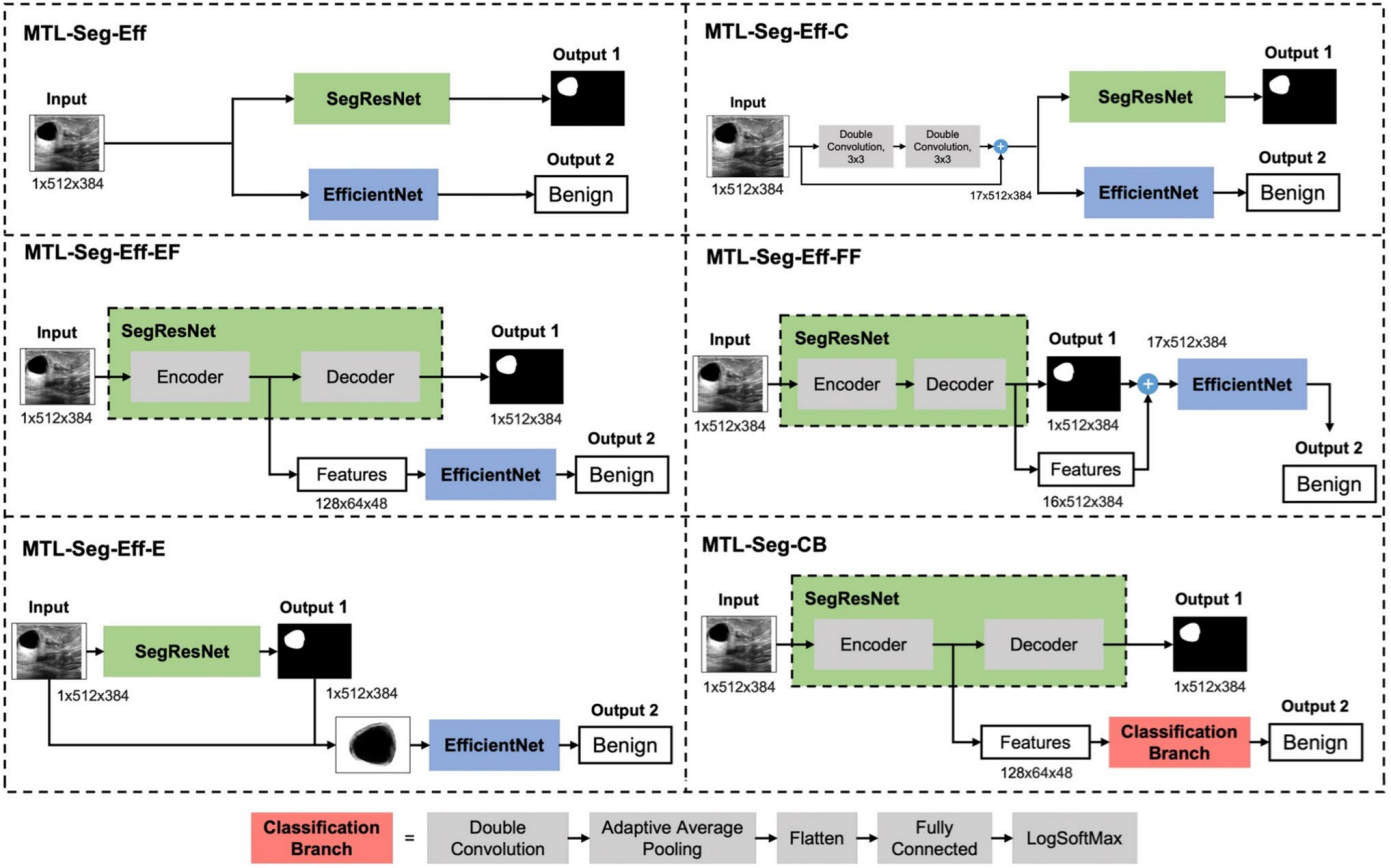
Overview of architectures for the MTL approach. MTL-Seg-Eff: parallel baseline; MTL-Seg-Eff-C: shared early convolutions; MTL-Seg-Eff-EF: EfficientNet using encoder features; MTL-Seg-Eff-FF: EfficientNet using decoder features and segmentation output; MTL-Seg-Eff-E: classification using segmentation-based ROI; MTL-Seg-CB: classification using encoder features

To promote and optimize performance for both segmentation and classification tasks, we designed and evaluated unified architectures where SegResNet and EfficientNet are trained simultaneously. In total, six different multi-task configurations were developed and tested in this study (Fig. 3). These architectures differ in how they connect the two networks and in the level of feature sharing between tasks, as described in Table 1, allowing an understanding of how architectural design and information sharing influence performance in MTL.

#### MTL with segmentation and classification branches in parallel (MTL-Seg-Eff)

In this parallel baseline architecture, SegResNet and EfficientNet are trained in parallel, with no explicit feature sharing between them. The original US image is simultaneously input into both networks, and each produces its respective output, namely the SegResNet generates a lesion segmentation mask, and EfficientNet outputs the classification. As training is performed simultaneously using a combined loss function, this configuration serves as a baseline for assessing task interaction.

#### MTL with shared initial feature extraction before parallel branches (MTL-Seg-Eff-C)

To promote feature sharing between the tasks, this architecture introduces a shared initial stage to the MTL-Seg-Eff architecture. Specifically, the input image first passes through two sets of double convolutional layers designed to extract feature maps, which are then concatenated with the original image and fed into the two task-specific branches. This design enables the model to leverage shared features in the early stages while preserving task-specific processing.

**Table 1:** Overview of the evaluated MTL architectures for breast lesion classification and segmentation

MTL architecture	Seg input	Cls input	Feature sharing	Main characteristics
MTL-Seg-Eff	Original image	Original image	None (parallel)	Baseline for task interaction, clean separation between tasks
MTL-Seg-Eff-C	Shared convolution features and image	Shared convolution features and image	Early shared layers	Promotes early coordination, reuses low-level features
MTL-Seg-Eff-EF	Original image	Encoder feature maps from SegResNet	Shared encoder	Uses high-level semantic features from the encoder
MTL-Seg-Eff-FF	Original image	Decoder features + predicted mask	Decoder-level and mask	Guides classification with shape and boundary features
MTL-Seg-Eff-E	Original image	ROI image based on predicted mask	Indirect (via mask)	Focus on lesion and surrounding tissue; reduces background
MTL-Seg-CB	Original image	Encoder feature maps from SegResNet	Shared encoder	Uses high-level semantic features from the encoder, lower complexity

#### MTL with classification using encoder feature maps from the segmentation path (MTL-Seg-Eff-EF)

In this configuration, EfficientNet is used to classify directly from the encoded features of SegResNet. Specifically, instead of using the raw image as input, EfficientNet receives intermediate feature maps extracted from the segmentation encoder. This approach enables EfficientNet to classify lesions based on high-level features learned during segmentation. Overall, this design encourages deeper feature sharing between tasks through the SegResNet encoder, while maintaining the architectural strengths of EfficientNet in the classification branch.

#### MTL with classification using decoder features and predicted segmentation mask (MTL-Seg-Eff-FF)

In this configuration, SegResNet processes the input image and produces both high-level feature maps from its decoder and a segmentation probability mask. For the classification branch, instead of using the raw image as input for classification, these SegResNet outputs are concatenated and fed into EfficientNet. Thus, the classification branch leverages segmentation-informed features, such as lesion shape, size, and boundary regularity, which are often closely associated with malignancy characteristics, enabling EfficientNet to make predictions guided by segmentation-driven features.

#### MTL with classification based on segmentation-enhanced ROI (MTL-Seg-Eff-E)

In this configuration, SegResNet also performs segmentation first, and the resulting mask is used to generate a ROI-based input image for classification. For that, the lesion segmentation is firstly dilated by 70% using a morphological operation with a disk-shaped structuring element. Afterward, the region inside the dilated mask is filled with the corresponding area from the original image, and the resulting image is then used as input to EfficientNet for classification. With this design, the classification branch evaluates an ROI image that incorporates both the lesion and its immediate surroundings, which are important regions for radiological diagnosis, while excluding irrelevant background regions. Thus, this setup enables the classification task to focus on meaningful anatomical context.

#### MTL with lightweight classification using encoder feature maps from the segmentation path (MTL-Seg-CB)

Similarly to the MTL-Seg-Eff-EF, this architecture uses SegResNet as a shared encoder for both tasks. However, instead of leveraging EfficientNet for classification, a simpler custom classification branch is added. This branch consists of a double convolutional layer, followed by adaptive average pooling, a flatten operation, a fully connected layer, and a LogSoftmax activation. This configuration was designed to assess the difference between using a lightweight classification branch versus a more complex architecture like EfficientNet.

### Training strategy

All proposed MTL configurations were trained using an end-to-end training approach, allowing both segmentation and classification branches to be optimized simultaneously. To ensure consistency and comparability across models, the same combined loss function was applied to all configurations, balancing the objectives of both tasks during the learning process. Specifically, for the classification task, the Cross-Entropy loss (1) was used, while for the segmentation task, the Dice Cross-Entropy loss (4) was applied. The latter combines the strengths of the Dice loss, which measures the similarity between the predicted and ground truth masks, with the Cross-Entropy loss, which provides stable gradients and encourages pixel-wise accuracy.

1 $${\mathcal{L}}_{\mathrm{C}\mathrm{E}\_\mathrm{c}\mathrm{l}\mathrm{a}\mathrm{s}\mathrm{s}}\left(\mathrm{p},\widehat{\mathrm{p}}\right)=-\frac{1}{\mathrm{C}}\sum_{\mathrm{c}=1}^{\mathrm{C}}\left({\mathrm{p}}_{c}\cdot \mathrm{log}\left({\widehat{\mathrm{p}}}_{c}\right)\right)$$

2 $${\mathcal{L}}_{\mathrm{D}\mathrm{i}\mathrm{c}\mathrm{e}\_\mathrm{s}\mathrm{e}\mathrm{g}}\left(\mathrm{y},\widehat{\mathrm{y}}\right)=1-\frac{2\sum_{\mathrm{n}=1}^{\mathrm{N}}({\mathrm{y}}_{\mathrm{n}}\cdot {\widehat{\mathrm{y}}}_{\mathrm{n}})}{\sum_{\mathrm{n}=1}^{\mathrm{N}}{{\mathrm{y}}_{\mathrm{n}}}^{2}+ \sum_{\mathrm{n}=1}^{\mathrm{N}}{{\widehat{\mathrm{y}}}_{\mathrm{n}}}^{2}}$$

3 $${\mathcal{L}}_{\mathrm{C}\mathrm{E}\_\mathrm{s}\mathrm{e}\mathrm{g}}\left(\mathrm{y},\widehat{\mathrm{y}}\right)=-\frac{1}{\mathrm{N}}\sum_{\mathrm{n}=1}^{\mathrm{N}}\left({\mathrm{y}}_{\mathrm{n}}\cdot \mathrm{log}\left({\widehat{\mathrm{y}}}_{\mathrm{n}}\right)\right)$$

4 $${\mathcal{L}}_{\mathrm{D}\mathrm{i}\mathrm{c}\mathrm{e}\mathrm{C}\mathrm{E}\_\mathrm{s}\mathrm{e}\mathrm{g}}={\uplambda}_{\mathrm{C}\mathrm{E}\_\mathrm{s}\mathrm{e}\mathrm{g}}\cdot {\mathcal{L}}_{\mathrm{C}\mathrm{E}\_\mathrm{s}\mathrm{e}\mathrm{g}}+{\uplambda}_{\mathrm{D}\mathrm{i}\mathrm{c}\mathrm{e}\_\mathrm{s}\mathrm{e}\mathrm{g}}\cdot {\mathcal{L}}_{\mathrm{D}\mathrm{i}\mathrm{c}\mathrm{e}\_\mathrm{s}\mathrm{e}\mathrm{g}}$$

In the equation (1), which denotes the Cross-Entropy loss for classification, $\mathrm{p}$ represents the ground-truth class distribution for the image, and $\widehat{\mathrm{p}}$ corresponds to the predicted probability distribution over class$\mathrm{c}$. For the remaining equations, $\mathrm{y}$ and $\widehat{\mathrm{y}}$ represent the ground-truth and the predicted result computed pixel-wise, respectively, where $\mathrm{N}$ denotes the number of pixels. The total loss of the proposed network is the weighted summation of these two individual task losses:

5 $${\mathcal{L}}_{\mathrm{T}\mathrm{o}\mathrm{t}\mathrm{a}\mathrm{l}}={\uplambda}_{\mathrm{D}\mathrm{i}\mathrm{c}\mathrm{e}\mathrm{C}\mathrm{E}\_\mathrm{s}\mathrm{e}\mathrm{g}}\cdot {\mathcal{L}}_{\mathrm{D}\mathrm{i}\mathrm{c}\mathrm{e}\mathrm{C}\mathrm{E}\_\mathrm{s}\mathrm{e}\mathrm{g}}+{\uplambda}_{\mathrm{C}\mathrm{E}\_\mathrm{c}\mathrm{l}\mathrm{a}\mathrm{s}\mathrm{s}}\cdot {\mathcal{L}}_{\mathrm{C}\mathrm{E}\_\mathrm{c}\mathrm{l}\mathrm{a}\mathrm{s}\mathrm{s}}$$

## Experiments

The proposed MTL architectures were evaluated and compared against each other and against their corresponding single-task models, namely SegResNet for segmentation and EfficientNet for classification. Additionally, the present study also included a comparative analysis with the state-of-the-art MTL architectures presented in [13–16]. Furthermore, a transformer-based multi-task architecture built upon the SwinUNETR [20] backbone was also implemented for comparison. These experiments are detailed in this section.

### Dataset

We evaluate the methods using one dataset compiled from two public 2D breast US images: BUSI [21] and UDIAT [22] datasets. The BUSI dataset, collected in the Baheya Hospital for Early Detection and Treatment of Women’s Cancer (Cairo, Egypt), comprises a total of 780 images, where 437 are benign lesions, 210 are malignant lesions, and 133 do not contain lesions. The images have an average size of 500 × 500, and the lesions vary in dimension and number. The UDIAT dataset was acquired at the UDIAT Diagnostic Center of the Parc Taulí Corporation (Sabadell, Spain), and it comprises 110 images of benign masses and 53 images of cancerous tumors. This dataset has a mean image size of 760 × 570, and most lesions are small. The two datasets were combined, but cases without lesions were not considered. Preprocessing was performed to uniformize the images by resizing the images and applying one-padding to maintain the original aspect ratio and obtain a final image size of 512 × 384. Finally, the images were normalized by changing the range of pixel intensity values to [0,1]. The final dataset comprises 810 images and respective ground truth segmentation tasks, where 547 are benign lesions, and 263 are malignant tumors. The data were randomly distributed as 80% for training, 10% for validation, and 10% for testing.

### Evaluation metrics

To evaluate the segmentation performance, the following metrics were employed: Dice Coefficient (DC), precision (*Pre*), sensitivity (*Sen*), and accuracy (*Acc*). Specifically, DC quantifies the overlap between the predicted and ground-truth masks, *Pre* measures the proportion of correctly identified positive pixels among all predicted positives, *Sen* reflects the model’s ability to correctly identify actual positive pixels, and *Acc* represents the overall proportion of correctly classified pixels in the entire image. To assess classification performance, F1-Score (*F1*), *Acc*, Area Under the Curve (AUC), and the Cohen’s kappa coefficient ($k$) were used. Here, *F1* provides a balance between precision and sensitivity, *Acc* indicates the proportion of correctly classified cases, AUC measures the model’s ability to distinguish between classes across different thresholds, and $k$ evaluates the agreement between predicted and true labels while accounting for chance agreement.

### Implementation details

The proposed methods and state-of-the-art networks evaluated for comparison were implemented in Python 3.9.7 and PyTorch 1.10.0, adapting the MONAI^1^ framework for the MTL networks, and trained on NVIDIA A100 40 GB GPU. For all the proposed MTL configurations, pre-trained weights of EfficientNet and SegResNet were used, enabling faster convergence and improved generalization. Regarding the literature MTL configurations, since no implementations were available, we reimplemented them using the provided representations and descriptions of the architectures. It should be noted that regarding the network proposed by Kang et al*.* [15], only the first stage was implemented, consisting of an MTL U-Net with a classification branch after the encoder, consisting of an adaptive middle clustering layer, a flat layer, an FC layer, and a softmax layer. Regarding the implemented transformer-based multi-task architecture (MTL-TR), this network employs SwinUNETR as the segmentation backbone. In this configuration, intermediate decoder features extracted from SwinUNETR are directly connected to a dedicated classification branch composed of convolutional layers, followed by global average pooling and fully connected layers. This design allows the classification task to leverage high-level representations learned by the transformer-based encoder–decoder structure, enabling joint optimization.

Each network was trained using the Adam optimizer with a learning rate of 0.0001 and a batch size of 10. The validation dataset was used for early stopping during training, while final model performances were evaluated on the independent testing set. During training, the images were shuffled and real-time data augmentation was applied to diversify the dataset and avoid overfitting while improving model performance. Multiple data augmentation techniques, including spatial and intensity-based transformations, were applied to efficiently generate diverse images. Spatial transformations included flips, zooming in, and grid distortion, whereas intensity-based transformations comprised intensity scaling and shifting, Gaussian noise and smooth addition, and contrast modification. Only combinations that produce images that resemble real US images were used. The weights of each loss of the total loss, ${\uplambda}_{\mathrm{D}\mathrm{i}\mathrm{c}\mathrm{e}\mathrm{C}\mathrm{E}\_\mathrm{s}\mathrm{e}\mathrm{g}}$ and ${\uplambda}_{\mathrm{C}\mathrm{E}\_\mathrm{c}\mathrm{l}\mathrm{a}\mathrm{s}\mathrm{s}}$, are set to 1 to contribute equally, aiming to promote equitable optimization across both segmentation and classification tasks. Regarding the Dice Cross-Entropy loss, to enhance the performance, the weight of ${\uplambda}_{\mathrm{D}\mathrm{i}\mathrm{c}\mathrm{e}\_\mathrm{s}\mathrm{e}\mathrm{g}}$ was increased, setting it to 0.7 and ${\uplambda}_{\mathrm{C}\mathrm{E}\_\mathrm{s}\mathrm{e}\mathrm{g}}$ to 0.3. These values ​​were obtained experimentally to obtain the best convergence of the model. To address the discrepancy in the number of cases among different classes, a balanced Cross-Entropy loss function is utilized. This approach ensures that each class receives equal consideration during training, promoting fair learning across all classes.

## Results

Table 2 presents the quantitative performance of the proposed MTL models, along with the corresponding results of the standalone SegResNet and EfficientNet baselines. To compare the proposed network designs with literature methods, Table 2 also presents the performance of the four state-of-the-art methods, as well as the transformer-based architecture. Among all configurations, MTL-Seg-Eff-EF achieved the best performance across all classification metrics, outperforming not only the other MTL architectures but also the individual EfficientNet classification model and the literature methods. In terms of segmentation performance, MTL-Seg-Eff-EF also outperformed the remaining MTL configurations, while maintaining results comparable to the individual SegResNet network.

**Table 2:** Quantitative comparison of the proposed methods for simultaneous segmentation (Mean ± Standard Deviation %, computed across the test samples) and classification (Mean %, computed across the test samples). Bold values indicate the best performance

Networks	Evaluation metrics							
	Segmentation				Classification			
	DC	*Pre*	*Sen*	*Acc*	*Acc*	AUC	*F1*	$k$
SegResNet	**81.47 ± 23.09**	84.16** ± **22.97	81.49 ± 23.84	**97.86 ± 2.89**	-	-	-	
EfficientNet	-	-	-	-	92.59	93.53	91.81	0.84
MTL-Seg-Eff	80.05 ± 24.10	83.37 ± 23.69	81.11 ± 24.96	97.68 ± 3.18	93.83	94.44	93.12	0.86
MTL-Seg-Eff-C	80.58 ± 23.22	83.62 ± 23.88	81.81 ± 21.50	97.36 ± 3.81	91.36	90.59	90.19	0.80
MTL-Seg-Eff-EF	81.19 ± 23.95	84.31 ± 23.81	81.59 ± 24.64	97.73 ± 3.28	**96.30**	**97.27**	**95.87**	**0.92**
MTL-Seg-Eff-FF	81.00 ± 23.12	83.15 ± 23.44	**82.92 ± 23.61**	97.61 ± 3.24	87.65	86.85	86.11	0.72
MTL-Seg-Eff-E	73.26 ± 24.83	84.11 ± 21.79	69.31 ± 27.48	96.65 ± 4.48	81.48	81.29	79.70	0.60
MTL-Seg-CB	80.74 ± 24.05	83.36 ± 24.02	80.97 ± 25.00	97.71 ± 3.05	90.12	92.73	89.41	0.79
Kang et al. [15]	81.10 ± 24.69	84.03 ± 24.46	81.39 ± 25.44	97.70 ± 3.52	90.12	88.67	88.67	0.77
Zhou et al. [13]	80.08 ± 26.54	84.32 ± 25.35	78.84 ± 28.15	97.71 ± 3.45	90.12	88.67	88.67	0.77
Chowdary et al. [16]	78.98 ± 26.88	79.86 ± 28.13	81.38 ± 25.83	97.14 ± 4.30	87.65	85.84	85.84	0.72
Le et al. [14]	76.85 ± 22.88	78.79 ± 25.15	78.36 ± 22.84	96.48 ± 4.94	86.42	85.94	84.86	0.70
MTL-TR [20]	80.51 ± 22.55	**87.18 ± 22.61**	78.66 ± 22.43	97.2 ± 94.00	85.19	84.02	83.33	0.67

To assess the statistical significance of the observed performance differences, statistical tests for both tasks were also conducted. For the segmentation task, the Wilcoxon signed-rank test was applied to compare each multi-task model against the SegResNet baseline. The results indicated no statistically significant improvements or degradation, except for the lowest-performing multi-task configuration (*p*-value < 0.05 for the MTL-Seg-Eff-E). For the classification task, McNemar’s test was used to compare each multi-task model against the EfficientNet baseline. Although some multi-task configurations achieved numerically higher performance metrics, the differences were not statistically significant.

**Fig. 4:**
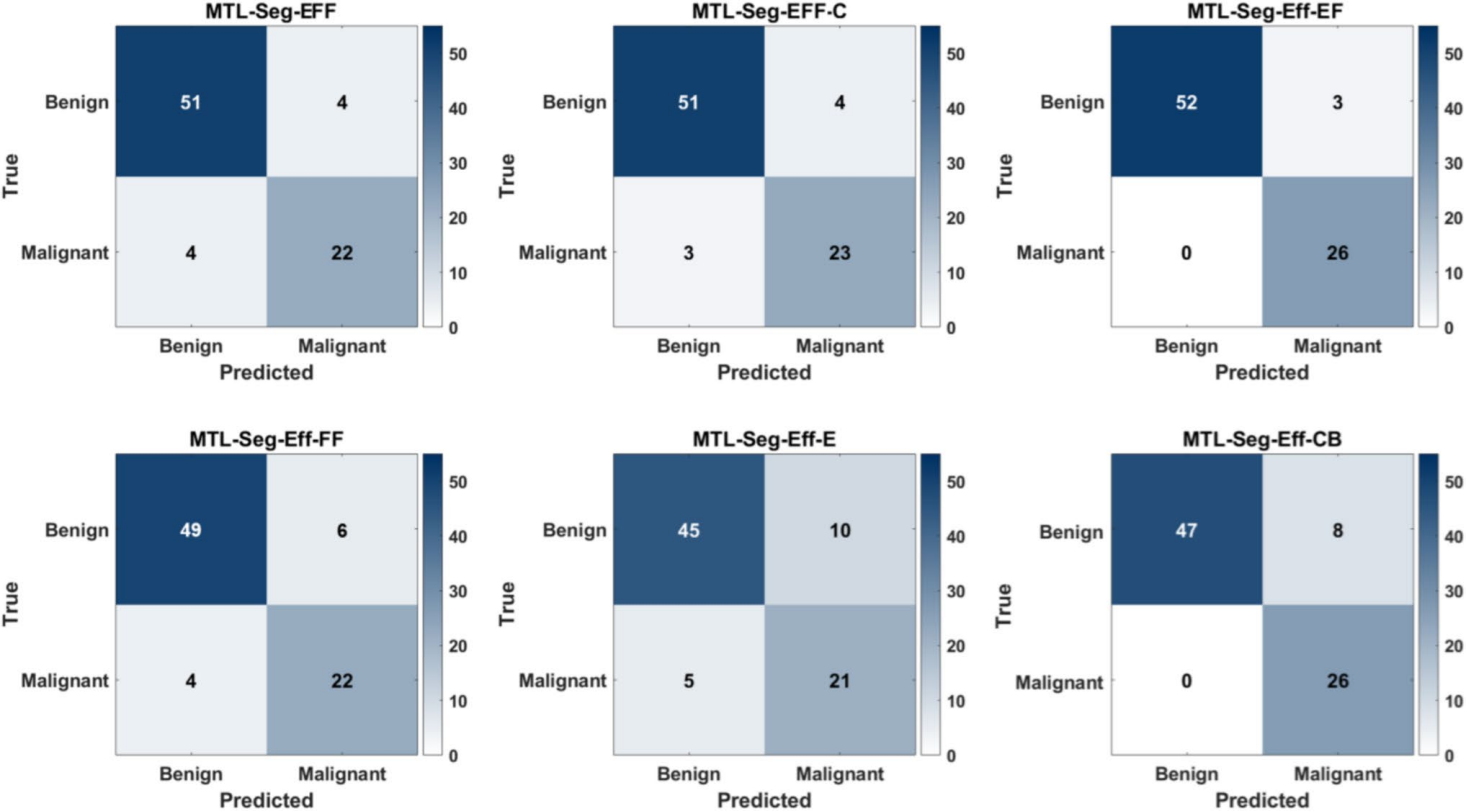
Confusion matrices illustrating the classification performance of the proposed MTL configurations

**Fig. 5:**
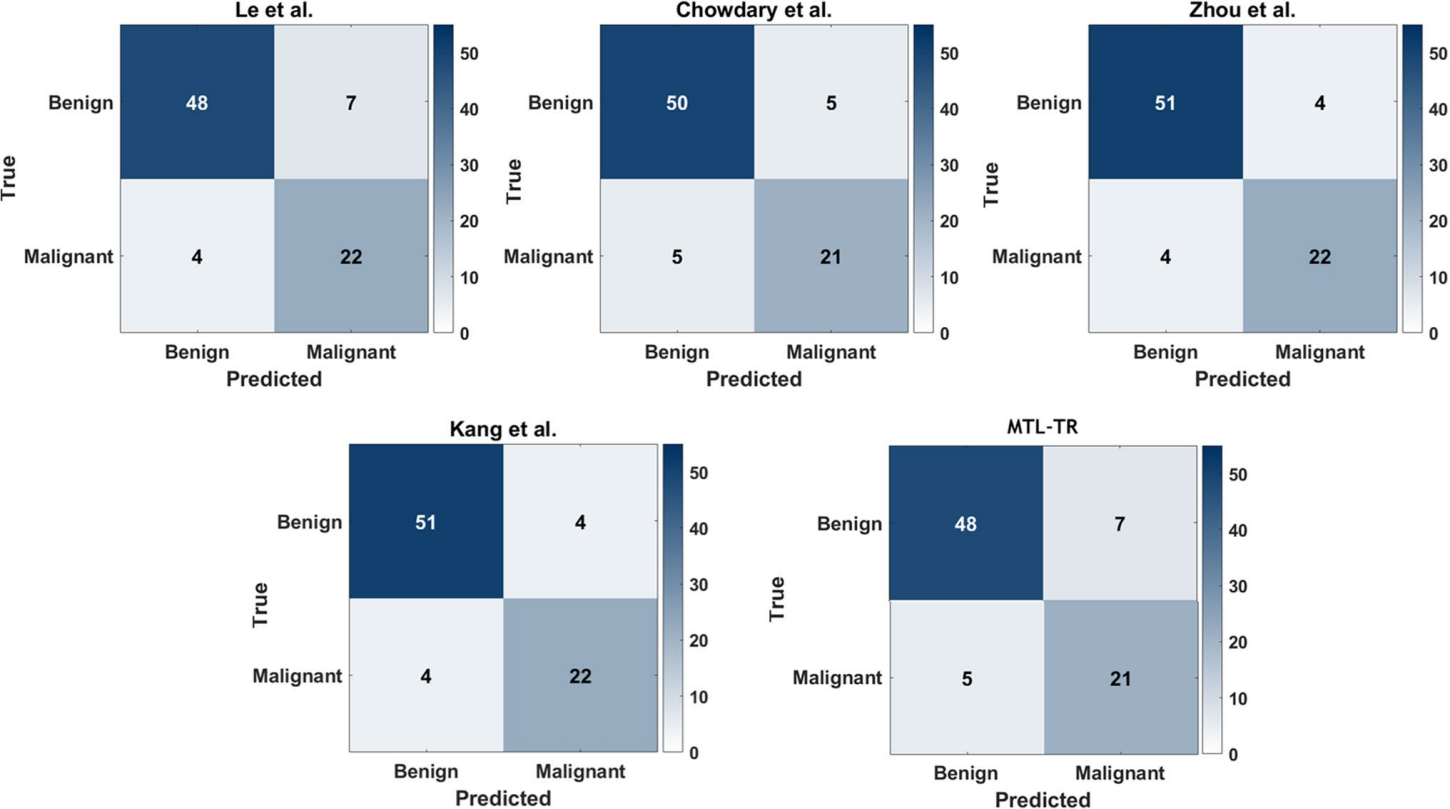
Confusion matrices illustrating the classification performance of the state-of-the-art networks

**Fig. 6:**
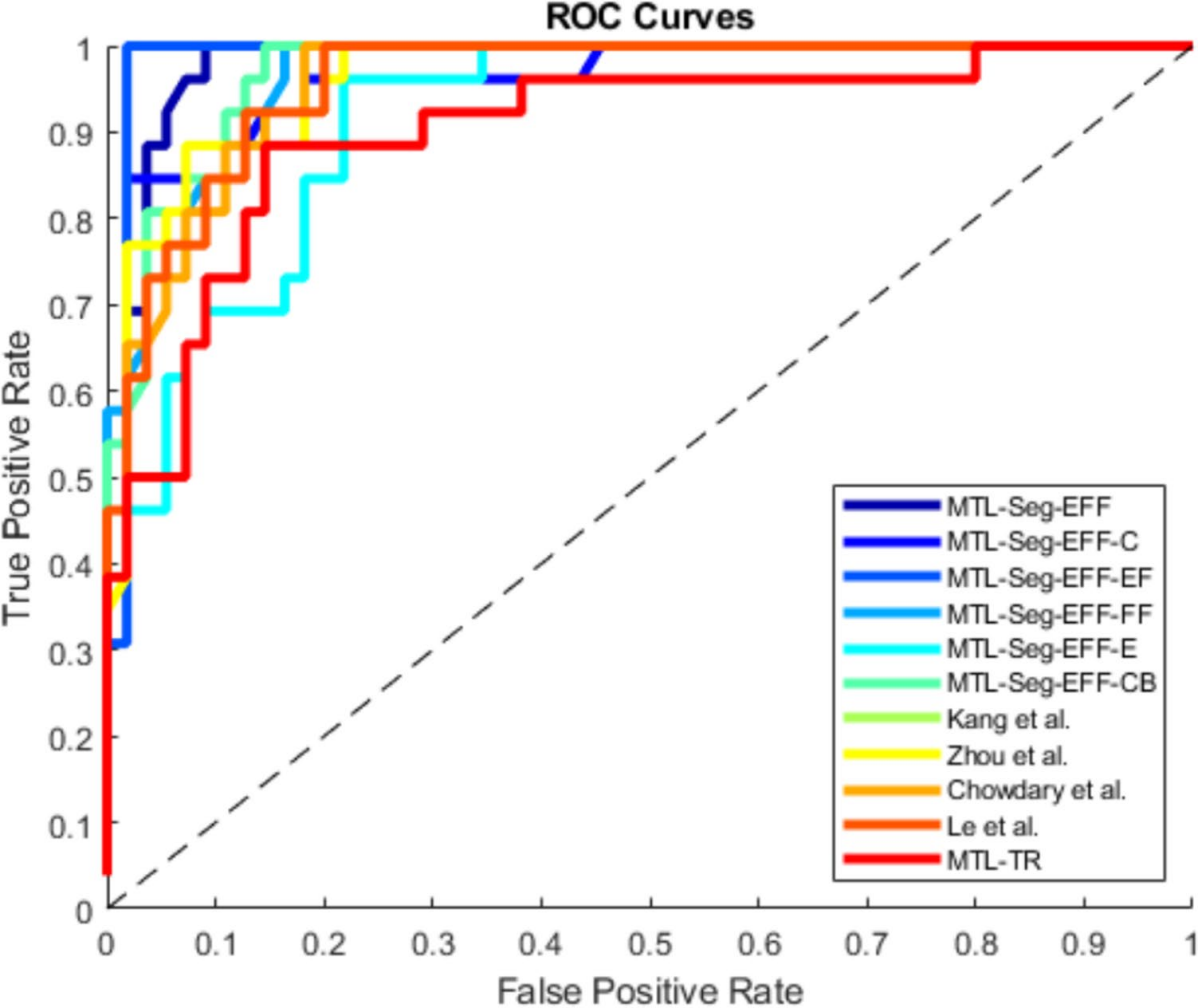
Receiver operating characteristic (ROC) curves for lesion classification using the proposed MTL architectures and state-of-the-art MTL methods

**Fig. 7:**
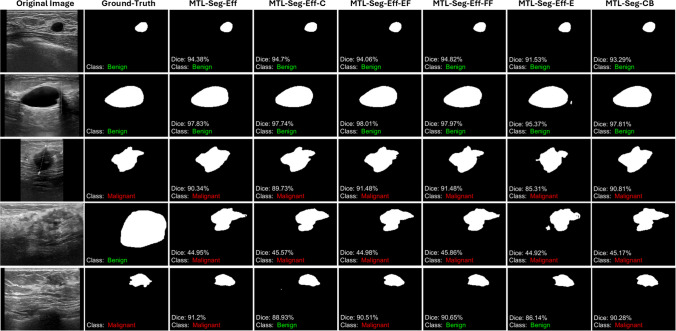
Visual results of different models for breast lesion segmentation and classification in US images

Figures 4 and 5 present the confusion matrices for lesion classification, to illustrate the classification performance in terms of correctly and incorrectly predicted benign and malignant lesions. Figure 4 shows the results for the proposed MTL architectures, while Fig. 5 displays the matrices for the state-of-the-art MTL methods. Additionally, Fig. 6 displays the receiver operating characteristic (ROC) curves of the classification approaches addressed in this study, validating that MTL-Seg-Eff-EF has high performance. The visual performance comparison of the different models for segmentation and classification of breast lesions in US is shown in Fig. 7.

## Discussion

This paper presented a study on different network architectures for the simultaneous segmentation and classification of breast lesions in 2D ultrasound images, leveraging MTL to optimize the performance of these heterogeneous yet interrelated tasks through shared feature representations. Training segmentation and classification simultaneously in a unified network aimed at enforcing one or both tasks, as they are highly related (e.g., lesion boundary characteristics or boundary artifacts can be used as a clue for classification and segmentation).

Six different network configurations combining a segmentation backbone (SegResNet) with a classification backbone (EfficientNet) were developed and evaluated on a large multi-center breast ultrasound dataset. Table 2 gives the quantitative performance comparison of these different models on breast lesion segmentation and classification. When analyzing the classification performance, the MTL-Seg-Eff-EF configuration, where the classification branch receives encoded features extracted from the shared encoder, consistently achieved the best results across all classification metrics, achieving a *F1* of 95.87%, an *Acc* of 96.3%, and an AUC of 97.27%. Moreover, when evaluating $k$-statistics, through Cohen’s kappa coefficient, it was found a $k$ value of 0.92, which demonstrates a strong agreement between the models’ predictions against the ground truth classifications, which can be corroborated by the confusion matrix in Fig. 4. The MTL-Seg-Eff-EF architecture enabled the classification network to operate on task-relevant features extracted during the segmentation process, rather than on raw image data. By leveraging these features, the network was able to capture high-level representations of crucial information such as lesion morphology, boundary characteristics, and local context, enabling the classification branch to more effectively distinguish between benign and malignant lesions. Thus, these findings support the hypothesis that deeper integration of segmentation-informed features into the classification pathway enhances diagnostic accuracy. Moreover, the MTL-Seg-Eff-EF configuration outperformed the state-of-the-art methods, the transformer-based architecture, and also the individual EfficientNet in the classification task. Although this improvement against EfficientNet did not reach statistical significance under the applied McNemar’s test, the numerical gains can suggest that the proposed multi-task approach effectively enhances classification performance.

When comparing the best-performing configuration with the remaining MTL architectures, a performance decrease was observed for the MTL-Seg-CB. Although both configurations share the same segmentation encoder and use its extracted features for classification, the MTL-Seg-CB adopts a lightweight custom classification head, whereas MTL-Seg-Eff-EF integrates a pre-trained, deeper classification network. These results suggest that the EfficientNet-based classifier is more robust to model complex patterns in the shared features, whereas a simpler classification head may not provide sufficient capacity to capture the necessary characteristics for accurate discrimination between benign and malignant lesions. A similar trend was observed for the transformer-based multi-task architecture. Although it outperformed the MTL-Seg-CB configuration, its classification performance did not reach the level achieved by the other models. This behavior may also be attributed to the relatively simple classification head attached to the transformer backbone. These results point to potential limitations in the capacity of lightweight architectures to effectively learn from high-dimensional features, particularly in complex medical image analysis tasks where subtle patterns distinguish classes. In opposite, deeper architectures, despite being more computationally demanding, may offer improved robustness. Thus, integrating a more expressive classification head within the multi-task frameworks could potentially enhance its discriminative capacity and improve overall classification performance. In this sense, this study highlights the importance of using not only effective feature sharing but also strong task-specific branches in multi-task learning setups.

When evaluating the simpler MTL configurations, namely MTL-Seg-Eff (parallel baseline) and MTL-Seg-Eff-C (parallel baseline with initial convolutional layers for shared processing), some interesting observations emerged. The MTL-Seg-Eff configuration, where the segmentation and classification branches operate independently from the raw image, achieved better classification performance than the individual EfficientNet and even outperformed the evaluated state-of-the-art MTL methods. However, surprisingly, the variant with initial shared convolutional layers before branching (MTL-Seg-Eff-C) did not yield improved results, obtaining lower performance than the baseline MTL-Seg-Eff. A possible explanation is that the shared initial layers failed to capture sufficiently informative features for both tasks simultaneously, not being these features maps enough to replace the original image information, and having also potentially introduced redundancy or noise. Additionally, the lack of depth in the shared layers could hinder the model’s ability to extract discriminative representations early in the network. Overall, these results suggest that simple early fusion may limit the model’s performance, whereas later sharing or more selective feature reuse might be more beneficial.

Finally, still analyzing the classification task, the MTL-Seg-Eff-FF (which uses decoder features and the predicted segmentation mask as input to the classification branch) and MTL-Seg-Eff-E (which builds an ROI image from the predicted mask to be used as input for the classification branch) achieved the lowest performance among the proposed configurations. Interestingly, in our previous study where different types of input were evaluated for EfficientNet, the ROI-based input yielded the best results [11]. This contrast suggests that the lower performance observed in the current study may be due to the use of predicted segmentation masks, instead of the ground-truth segmentations used in [11], which introduces a dependency on the quality of the segmentation output. When the segmentation is not highly accurate, the classification input becomes less reliable, potentially impairing the model’s ability to distinguish between benign and malignant lesions. Therefore, these architectures appear to be more sensitive to segmentation errors, which can negatively affect overall classification performance. To further investigate this dependency, a sample-level descriptive analysis was performed for these two architectures. Specifically, Dice scores were compared between correctly and incorrectly classified samples. For MTL-Seg-Eff-FF, correctly classified samples achieved a mean Dice of 81.73%, whereas misclassified samples presented a lower mean Dice of 76.63%. Similarly, for MTL-Seg-Eff-E, the mean Dice was 73.44% for correctly classified cases and 72.36% for misclassified cases. Although the differences are moderate, incorrectly classified samples exhibited lower Dice values, suggesting that lower segmentation quality tends to be associated with misclassification. These observations highlight the potential risks of error propagation in MTL frameworks, particularly when later tasks rely on imperfect intermediate outputs. Such cascaded architectures may be more vulnerable to small segmentation inaccuracies, and therefore, consistency mechanisms should be applied to mitigate segmentation errors.

Concerning the segmentation results, it was observed that, unlike the classification task, segmentation performance did not show significant improvements over the individual SegResNet model, as confirmed by the Wilcoxon signed-rank tests. The best-performing MTL configurations achieved results that were comparable to the individual segmentation network, but without notable gains. This suggests that, within the multi-task learning framework, the classification task benefits more from the joint training setup. This is likely because segmentation inherently provides valuable spatial and structural information, such as lesion boundaries, shape, and location, that can directly support and guide the classification task. In contrast, classification does not inherently enhance the spatial precision required for accurate segmentation. Therefore, while segmentation helps classification by narrowing the focus to clinically relevant regions, the inverse benefit appears limited, resulting in asymmetric task gains within the MTL setup. Moreover, in MTL setups, shared representations might prioritize the task that dominates the gradients or has a stronger influence during training, in this case, classification, leading to less performance gain for segmentation.

To visually assess the performance of the MTL architectures, Fig. 7 presents five representative examples from the test set. The first and second rows illustrate a benign lesion that was successfully segmented and classified by all methods. This lesion displays typical benign features, such as smooth, oval-shaped contours, clear distinction from the background tissue, and posterior acoustic enhancement. The third row shows a malignant lesion, which was also correctly segmented and classified by all models. This lesion exhibits evident malignant characteristics, including an irregular shape, non-parallel orientation, spiculated margins, and posterior acoustic shadowing, making it easier for the models to identify and correctly classify. The fourth image shows a lesion that all methods failed to classify and segment. In fact, although the ground truth labels the lesion as benign with an oval-shaped segmentation, the lesion itself appears heterogeneous and irregular, which likely contributed to the misclassification and poor segmentation performance across all models. Finally, the fifth example illustrates a challenging case involving a malignant lesion that a few methods failed to classify correctly. Although the lesion presents irregular boundaries, which is a typical indicator of malignancy, certain regions display smoothed margins. This combination may have misled some models, which produced smooth and rounded segmentation contours and misclassified the lesion as benign. Thus, the presented examples demonstrate a clear relationship between the segmentation and classification tasks, as the performance of segmentation has a direct impact on the performance of the classification. Specifically, when the lesion is poorly segmented, the classification model is unable to accurately classify the lesion.

Overall, our experimental results demonstrated the added value of MTL to optimize highly related tasks such as lesion segmentation and classification. Particularly, the findings of this work have practical clinical implications. From a diagnostic perspective, boosting classification performance is particularly valuable, as it directly impacts lesion malignancy detection. The ability to improve classification without compromising segmentation suggests that MTL can reduce diagnostic errors while maintaining spatial interpretability. Additionally, the high agreement levels with radiologist annotations strengthen the clinical relevance of the model’s predictions, potentially supporting decision-making in settings with limited expert availability. Therefore, the proposed MTL networks, particularly the best-performing architecture, demonstrate promising applicability in real-world clinical practice.

Although the dataset includes images from two independent centers, which introduces a degree of acquisition variability, the overall sample size remains relatively limited. As such, the limited number of centers and images represents a limitation of this study. Therefore, to further study, validate, and refine the proposed MTL configurations, training on a larger and more diverse dataset is essential. Future work will focus on acquiring a larger multi-center dataset to corroborate the superiority of the best-performing MTL architecture. Moreover, this dataset will be used to improve the performance, generalization, and robustness of the model, making it more reliable for use in clinical practice. Additionally, a similar study is planned for 3D breast US imaging, which offers several advantages over 2D images, including improved lesion localization, richer spatial context, enhanced feature extraction, reduced ambiguity, and greater robustness to variability. These benefits could enable the development of stronger segmentation and classification models. Lastly, it is intended to incorporate intra- and inter-task consistency learning approaches in the MTL configurations to enforce coherent predictions across multiple related tasks, further boosting the accuracy and reliability of the segmentation and classification framework.

## Conclusion

In this study, different MTL architectures were developed and applied for simultaneous breast lesion segmentation and classification on a multi-center breast US image dataset. The experimental results demonstrated that a configuration combining the segmentation network with a classification branch processing shared encoded features from the segmentation branch achieved the best overall performance. This configuration significantly improved classification results compared to the individual EfficientNet and outperformed other state-of-the-art MTL approaches from the literature. Overall, the proposed study highlights the potential of carefully designed MTL strategies in enhancing diagnostic performance by effectively leveraging task synergy and feature sharing. Future work will focus on further optimizing task interactions, integrating task-interdependency loss functions, and validating the proposed architectures on larger and more diverse clinical datasets.

## Data Availability

The code for the proposed multi-task network configurations is available at https://github.com/2Ai-Laboratory/Breast_MTL.

## Footnotes

1 https://github.com/Project-MONAI/MONAI
